# Supplementary material for: A Humanized Yeast Phenomic Model of Deoxycytidine Kinase to Predict Genetic Buffering of Nucleoside Analog Cytotoxicity
Source: Genes (Basel). 2019 Sep 30;10(10):770. doi: 10.3390/genes10100770 (PMC6826991; doi:10.3390/genes10100770)
Supplement: Supplementary file 1 [file genes-10-00770-s001.zip › Additional_Files/Additional_File7_GOTermHeatmaps.zip!/Additional_File7_GOTermHeatmaps/protein_deubiquitination.pdf]

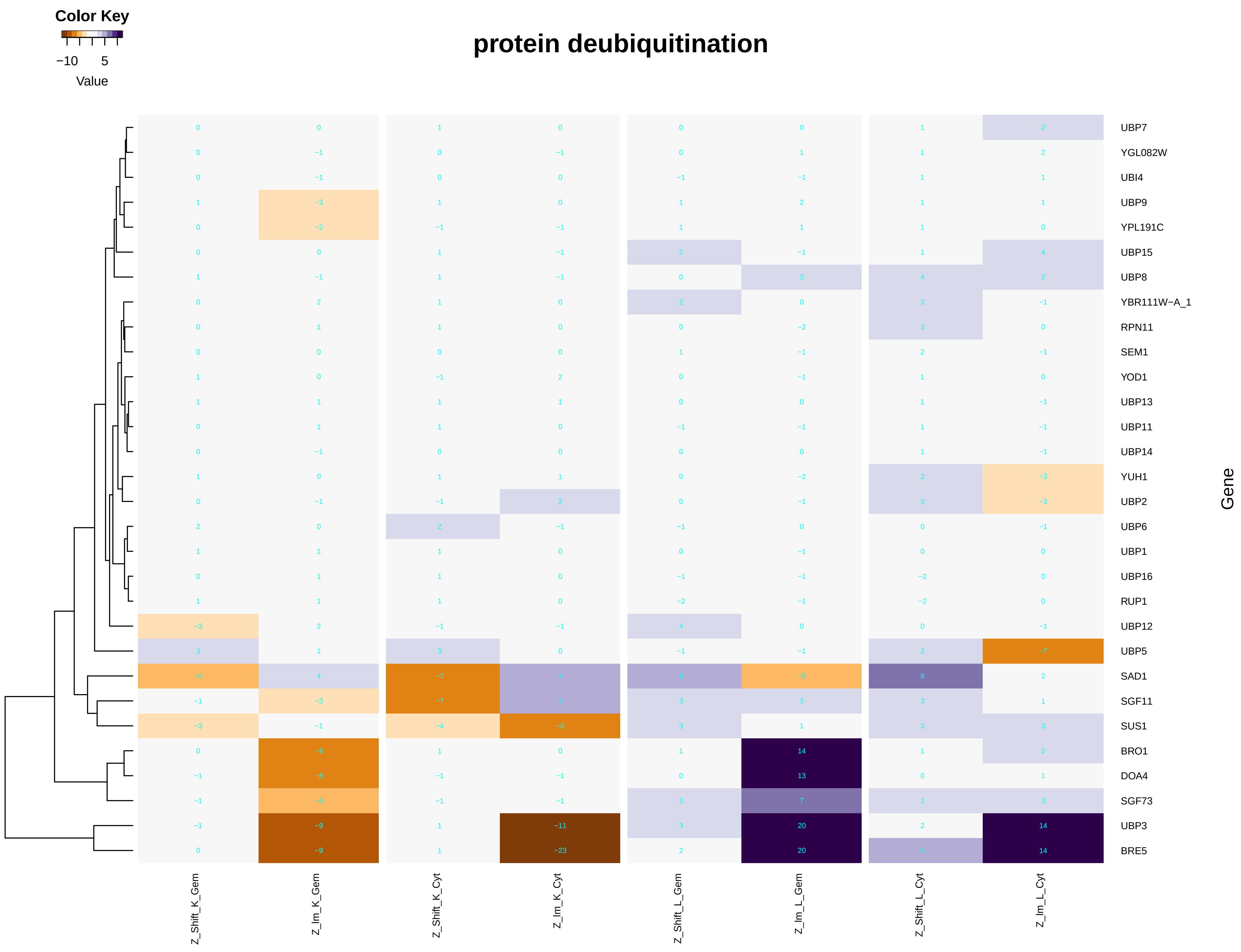
protein deubiquitination
Color Key
−10
5
Value
Gene
UBP7
YGL082W
UBI4
UBP9
YPL191C
UBP15
UBP8
YBR111W−A_1
RPN11
SEM1
YOD1
UBP13
UBP11
UBP14
YUH1
UBP2
UBP6
UBP1
UBP16
RUP1
UBP12
UBP5
SAD1
SGF11
SUS1
BRO1
DOA4
SGF73
UBP3
BRE5
Z_Shift_K_Gem
Z_Im_K_Gem
Z_Shift_K_Cyt
Z_Im_K_Cyt
Z_Shift_L_Gem
Z_Im_L_Gem
Z_Shift_L_Cyt
Z_Im_L_Cyt

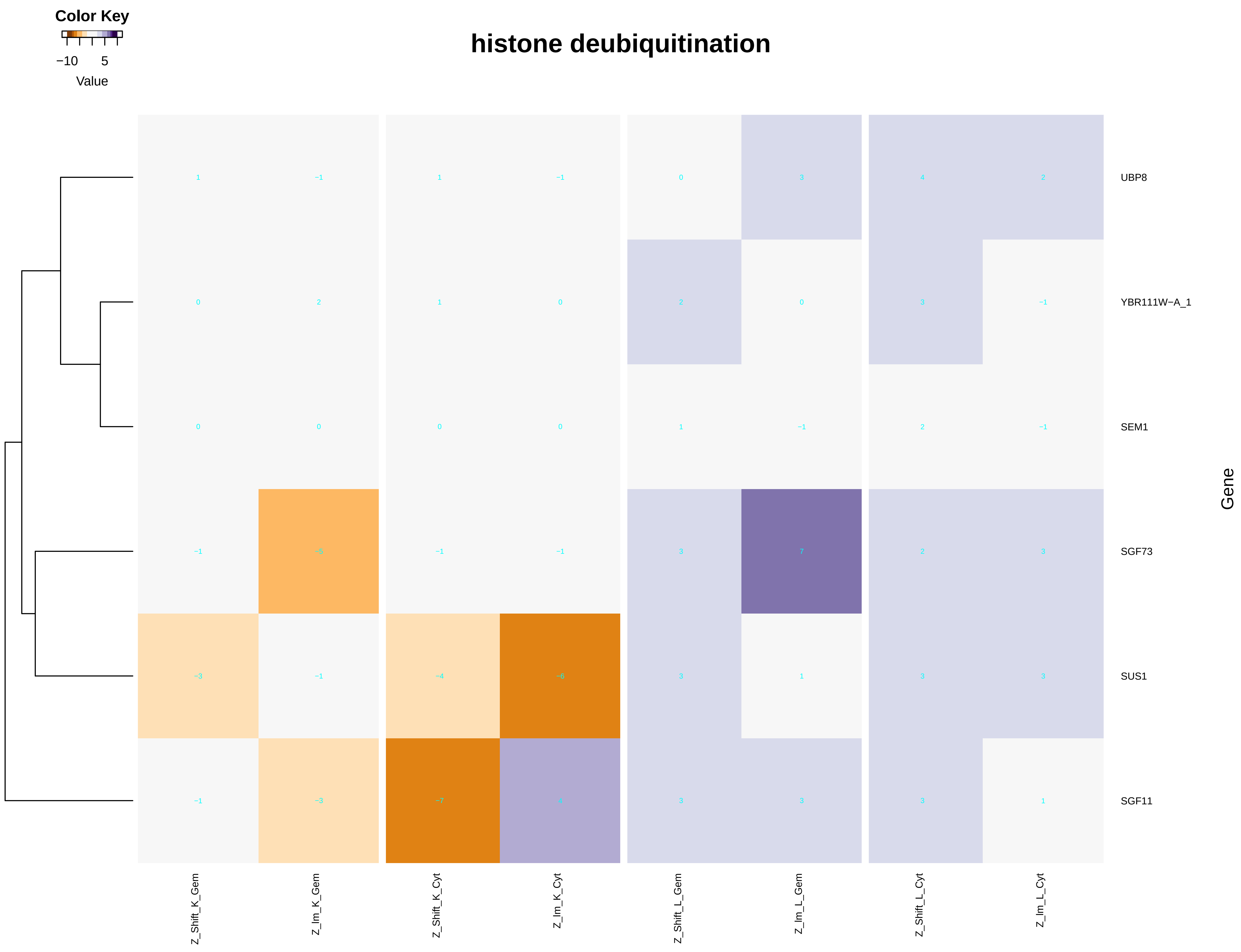
Color Key
−10
5
Value
histone deubiquitination
UBP8
YBR111W−A_1
SEM1
SGF73
SUS1
SGF11
Gene
Z_Shift_K_Gem
Z_Im_K_Gem
Z_Shift_K_Cyt
Z_Im_K_Cyt
Z_Shift_L_Gem
Z_Im_L_Gem
Z_Shift_L_Cyt
Z_Im_L_Cyt
